# Artificial Intelligence applications in Advance Care Planning

**DOI:** 10.1093/geroni/igaf122.3389

**Published:** 2025-12-31

**Authors:** Girish Hemrajani, Debra Dobbs

**Affiliations:** University of South Florida, Tampa, Florida, United States; University of South Florida, Tampa, Florida, United States

**Keywords:** Artificial intelligence, advance care planning, health equity, predictive analytics, natural language processing.

## Abstract

This scoping review examines artificial intelligence (AI) applications in advance care planning (ACP), synthesizing evidence from databases like PubMed, Web of Science, and Scopus (2019-2024), ten articles, using PRISMA guidelines. The review focused on AI tools for ACP documentation, decision-making, and workflow optimization, excluding studies lacking clinical validation or patient-centered outcomes. Findings reveal promising AI applications in ACP: predictive models, like Stanford’s EHR-based mortality predictor, improved high-risk patient identification, triggering earlier ACP discussions. Natural Language Processing reduced ACP documentation review time significantly. AI-powered chatbots increased ACP literacy among older adults by 40%, addressing health disparities. Challenges like algorithmic bias from non-diverse datasets exacerbates inequities in ACP access persist. Clinician resistance stems from unclear liability frameworks and poor EHR interoperability. Only 25% of tools address caregiver burden or surrogate decision-maker needs. Research gaps include limited evidence on AI-driven e-planning tools’ cost-effectiveness, lack of standardized metrics for evaluating AI’s impact on patient autonomy and understudied ethical risks of over-reliance on AI for sensitive end-of-life conversations. Future directions focus on equity-driven design, the integration of e-planning into electronic medical records, and the assessment of AI’s capacity to alleviate caregiver burden and enhance preparedness of surrogate decision-makers. This research emphasizes the necessity to transform ACP from a documentation-oriented process to a communication-centered continuum, utilizing AI to democratize access and tackle systemic inequalities. It highlights the potential of AI to transform ACP, while emphasizing the importance of ethical implementation and patient-centered outcomes.

